# Evaluation of ERG Expression in Prostate Adenocarcinoma and Its Prognostic Impact in Patients Survival Rate

**DOI:** 10.30699/IJP.20201.530515.2644

**Published:** 2021-07-06

**Authors:** Hedieh Moradi Tabriz, Leila Aghapour Sabaghi, Amirreza Nabighadim, Elham Elham, Seyed Mohammad Kazem Aghamir

**Affiliations:** 1 *Department of Pathology, Sina Hospital, Tehran University of Medical Sciences, Tehran, Iran*; 2 *Pediatric Urology and Regenerative Medicine Research Center, Children’s Medical Center, Tehran University of Medical Sciences, Tehran, Iran*; 3 *Urology Research Center, Tehran University of Medical Sciences, Tehran, Iran*

**Keywords:** ERG expression, Survival, Prognosis, Prostate Cancer

## Abstract

**Background & Objective:**

Some certain markers, including prostatic specific antigen (PSA), are being used to screen prostate cancer (PC), but none of them have sufficient sensitivity and specificity for evaluation of prognosis. Currently, genetic variants have found their place in the prognosis of PC. ETS-related gene (ERG) expression and its intensity have contradictory evidence regarding ERG expression with PC incidence or associating outcome. Our purpose was to survey the relationship of ERG expression and its intensity with PC and relative clinical outcome.

**Methods:**

We studied the immunohistochemichal (IHC) expression of ERG in 101 radical prostatectomy specimens with PC of different histologic grades. All samples were chosen from pathology department of Sina hospital in Tehran-Iran from 2011 to 2018. Positive ERG expression and its association with Gleason score, preoperative PSA, metastasis status, stage and grade of tumors was evaluated.

**Results:**

In total, ERG expression was observed in 42 cases (41.58%) and of these, 7 (16.66%) were categorized as weak, 13 (30.95%) moderate and 22(52.38%) as strong. There was no significant correlation between ERG expression and age, preoperative PSA, Gleason score, lymph node involvement, metastatic pattern, stage, and grade of the tumor (*P*>0.05). ERG expression frequency in the two groups of survived and expired patients was 42.85% and 0%, respectively; despite the noticeable difference, it was not statistically significance (*P*=0.264).

**Conclusion:**

Evaluation of ERG expression and its intensity may have no essential role as an acceptable prognostic factor in Iranian’s population for anticipating whether PC itself or the outcomes accompanied. This relation is vigorously under the influence of geographical/ethnical features.

## Introduction

Prostate adenocarcinoma is known as the second predominant cancer in the male population of developed countries like United States (1), with a widely higher prevalence among western communities (2). Approximately more than 240 thousand of new cases are diagnosed annually (3), including 16% of all men with the age of 67 years and older. The mortality rate of Prostate Cancer (PC) in the US was almost 30 thousand in 2012, occupying the second place between the cancers after lung cancer (4). Nowadays, some various chemical markers, including prostatic specific antigen (PSA), are being used to screen PC; however, none of these markers have sufficient sensitivity and specificity for prognosis evaluation (5, 6). So, it is essential to discover the factors, which play an important role affecting PSA level. Studies showed that prostatitis can increase PSA level. The age of these patients is usually high and might need colonoscopy for other reason which colonoscopy can increase the PSA level. So, it is required to question for a colonoscopy before measuring PSA (7). But, cystoscopy has no effect on PSA level and does not lead to its elevation (8). Despite the widespread application of PSA in the screening of PC, this neoplasia continues to immolate a huge number of victims; furthermore, its value in the early and seasonable prognosis of cancer has been cast doubt on (6). The high mortality rate of PC mainly is due to disease relapse and also progression to a meta-static disease; hence, detection of markers and specific methods for diagnosis and even treatment follow-up remains a necessity (9, 10). In this regard and for the first time in 2005, ERG (ETS-related gene) was introduced as a particular proto-oncogene for PC and has been demonstrated increased expression in more than 72% of PC cases (11). ERG, a member of the ETS (erythroblast transformation-specific) family, located on Chromosome 21, serves as a transcription regulator, which innately plays a considerable role in embryonic development, cell proliferation, differentiation, angio-genesis, inflammation, and apoptosis (12, 13). 

In the preliminary studies, ERG or ETS related products have been showed to be fundamental in hematopoiesis, hematopoietic stem cells normal function and also maintaining the platelet regular function and count (14). Additionally, ERG expression has been investigated in PC; the most common genetic alteration is the fusion of 5´ non-coding region of TMPRSS2 gene with one of the ETS family members; ERG involves in more than 90% of these fusions so can be tracked by immunohistochemistry (IHC) (15, 16). TMPRSS2 in prostate tissue associates with decreased expression and function of androgens (6). In the cultured prostate cancerous cells, androgen dependent ERG up-regulation in the Vertebral-Cancer of the Prostate (VCaP) androgen-sensitive cell lines has been detected (17). Overall, based on accomplished studies, TMPR-SS2-ERG fusion isoforms have been propounded as the PC progression mediator (18). It should be noted that there is a crucial difference in PC prevalence amongst western and eastern countries (19). PC incidence in western communities is 20 times higher than in eastern states. Additionally, prior studies demonstrated that TMPRSS2 and ERG fusion in the east, unlike the west, had far less frequency (21% in Korea and 28% in Japan, in comparison to 42 to 60% in USA) (20). These variants and geographical/ethnical differences are possibly linked to distinction in various molecular mechanisms in prostate tumorigenesis in the noted countries. 

There are two important questions coming in the mind we would like to know; first about the frequency of positivity of this marker in astern societies as well as the diagnostic value in the early stages of PC; and second, to to detect any association between expression of this indicator and the consequences of disease such as mortality and survival rate in long term. Therefore, the present study initially evaluated the ERG expression in a selected group of Iranian's population; then the prognostic value of the obtained data in the survival rate prediction was examined.

## Material and Methods

Patients

In the current retrospective cohort study, we included 101 patients with PC which had undergone radical prostatectomy between 2011 and 2018. All the patients were selected from electronic registry of Department of Pathology, Sina hospital affiliated to Tehran University of Medical Sciences. Sample size was calculated using Formula 1 with confidence level 95% and α is 0.05. 

$x=\frac{\mathrm{P*}\left(1-P\right)*Z^{2}1-\alpha /2}{\mathrm{d²}}$                     Formula 1

The following data including demographic information, pre-operation PSA, Gleason score, the extent of tumor invasion, tumor's grade and stage, lymph node involvement, distant metastasis, and formalin-fixed paraffin-embedded primary tumor samples for IHC analysis were considered as requirements for enrollment of each patient. The duration of follow-up was calculated from the date of surgery to the date of death or 12 months. So, all the patients were evaluated after 12 months from surgery for recurrence or distant metastasis. Cases with incomplete information were excluded. The study protocol was approved by the Ethics Committee of Tehran University of Medical Sciences (ethical code: IR. TUMS.MEDICINE.REC.1398.004).

ERG Expression

**Fig. 1:**
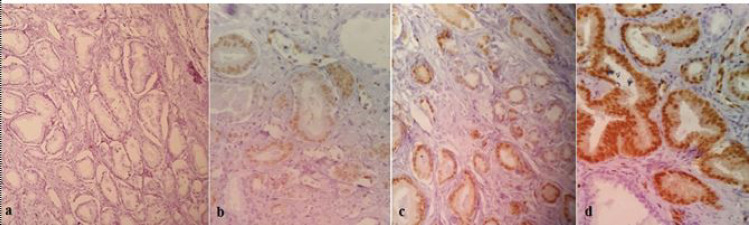
Different ERG expression intensity. a: negative expression; b: weak expression; c: moderate expression; d: strong expression

For Immunohistochemical staining to evaluate the ERG gene expression (mouse monoclonal, Biocare Medical company, clone 9FY), 3 microns' sections were obtained from every patient's appropriate paraffin block in the pathology ward. Each section was placed 20 minutes at 58ºC. Afterward, the sections were deparaffinized and rehydrated by the aid of xylene bath and graded alcohol series. Phosphate buffer was added to peroxidase inactivation. Slides were then incubated with the primary antibody at room temperature for one hour. After washing with PBS, secondary antibody was added, which was washed after 30-40 minutes. Slides were counterstained with hematoxylin. ERG protein has a nuclear expression and on the other hand, its cytoplasmicdiffusion is weak. The prepared slides were observed and examined by two pathologists and IHC results were categorized into four groups, including negative (-), weak (1+), moderate (2+) and strong (3+). Similarly, ERG gene expression was assessed in non-tum-oral tissues, too (Figure 1). Endothelial cells of small vessels and the lymphocytes serve as positive internal controls (21). 

Statistical Analyses

Parametric data were presented as mean and standard deviation (mean ± SD) and nonparametric ordinal data were presented as frequency and relative frequency. T-test and Chi-square test were performed on quantitative and qualitative data, respectively. ERG prognostic value in predicting patients' survival and the mortality rate were determined by a Cox proportional hazards test and the Kaplan-Meier method. The P-value less than 0.05 was considered statistically significant. For statistical analyses of the data, SPSS software version 21 (SPSS Inc., Chicago, Ill., USA) was used.

## Results

Basic Information

Among 101 prostate cancer patients enrolled in this study, the average age was 64.81±7.11 ranging from 51 to 81. As illustrated in Table 1, the mean Gleason score was 7.57±1.34. Regarding the pathologic tumor stage, PT1a in 2 (1.98%), PT1b in 1(0.99%), PT2a in 17 (16.83%), PT2b in 10 (9.9%), PT2c in 43 (42.57%), PT3a in 6 (5.94%), PT3b in 20 (19.8%) and PT3c was reported in 2 (1.98%) of cases. Lymphatic involvement and distant metastases were present in 5 (4.95%) and 7 (6.93%) samples, respectively. Of 101 specimens, 25 (24.75%) took place in in grade group 1, 40 (39.6%) in grade group 2, 16 (15.84%) in grade group 3, 4 (3.96%) in grade group 4 and 16 (15.84%) grade group 5. The mean amount of primary preoperative PSA was 15.77±13.5. In terms of cancer-related mortality status during the performed follow-up, 3 (2.97%) of the patients passed away; but, the cause of death was perioperative problems in the first case, comorbid esophageal cancer for the second one and in the third patient was indeterminate. The overall survival rate was 97%.

**Table 1:** Primary characteristics of the patients with prostate cancer

Relative Frequency	Frequency	Mean ± SD		
---	---	64.81±7.11	**Age**	
---	---	7.57±1.34	**Gleason score**	
---	---	15.77±13.5	**Primary PSA Level**	
**1.98%**	2	---	**PT1a**	**Tumor Stage**
**0.99%**	1	---	**PT1b**	
**16.83%**	17	---	**PT2a**	
**9.90%**	10	---	**PT2b**	
**42.57%**	43	---	**PT2c**	
**5.94%**	6	---	**PT3a**	
**19.80%**	20	---	**PT3b**	
**1.98%**	2	---	**PT3c**	
**100%**	101	---	**Total**	
**4.95%**	5	---	**Lymph node involvement**	
**6.93%**	7	---	**Metastasis**	
**24.75%**	25	---	**1**	**Tumor Grade Group**
**39.60%**	40	---	**2**	
**15.84%**	16	---	**3**	
**3.96%**	4	---	**4**	
**15.84%**	16	---	**5**	
**100%**	101	---	**Total**	

ERG Expression Depending on Primary Specificities and Disease Outcome 

We identified a total of 42 cases (41.58%) of ERG expressed samples; 7 (16.66%) out of those were classified as weak expression while 13 (30.95%) and 22 (52.38%) were as moderate and strong, respectively. . Patients’ average age and mean Gleason score in positive and negative expression group were 64.24±7.71, 65.22±6.69, and 7.88±1.89, 7.36±1.74, respectively; according to the given data, there was no significant relation between ERG expression and age, mean and every state of Gleason score (*P*=0.497, *P*=0.152 and *P*=0.211). 

The frequency of ERG expression in different tumor grades and stages are displayed in Table 2 in which no significant differences in expression rate were seen in any of the grades and stages of tumors (*P*=0.066 and *P*=0.462). ERG expression was observed in 40% of all lymph node positive samples and in 41.66% of negative lymph nodes specimens; however, no significant correlation was detected (*P*=0.926). 

ERG expression was seen in 42.85% of metastatic and 41.48% of non-metastatic cases, indicating no considerable difference (*P*=0.944). According to the data provided in Table 2, average PSA level and ERG expression were not statistically correlated (*P*=0.964). Despite the phenomenal difference of ERG expression in two groups of expired and survived patients, there was no remarkable statistical association (*P*=0.264). 

ERG Expression Intensity Depending on Primary Specificities and Disease Outcome

All the data concerning primary specificities and disease outcome corresponding to ERG expression intensity was provided in Table 3 and also comparisons addressing in this section was among these three groups. Regarding the close numbers of mean ages and Gleason score in aforementioned groups, no considerable affinity was established (*P*=0.696 and *P*=0.493). Referring to the given percentages in Table 3, we were incapable of finding a notable relation between different tumor stages and grades and ERG expression intensity (*P*=0.402 and *P*=0.547). Similarly, the same thing was verified for the lymph node involvement (*P*=0.385), metastasis status (0.168) and mean PSA level (*P*=0.329). Eventually, as ERG had no expression in expired patients, evaluating the expression intensity association with mortality was not feasible.

**Table 2:** ERG expression depending on primary characteristics

P-value		Relative Frequency		Frequency		Mean ± SD			
		- ERG expression	+ ERG expression	- ERG expression	+ ERG expression	- ERG expression	+ ERG expression		
**0.497**		---	---	---	---	65.22±6.69	64.24±7.71	**Age**	
**0.152**		---	---	---	---	7.36±1.74	7.88±1.89	**Gleason score**	
**0.964**		---	---	---	---	15.83±13.84	15.70±13.22	**Primary PSA**	
**0.462**		3.38%	0	2	0	---	---	**PT1a**	**Tumor Stage**
		1.69%	0	1	0	---	---	**PT1b**	
		20.33%	11.9%	12	5	---	---	**PT2a**	
		10.16%	9.52%	6	4	---	---	**PT2b**	
		38.98%	47.61%	23	20	---	---	**PT2c**	
		6.77%	4.76%	4	2	---	---	**PT3a**	
		18.64%	21.42%	11	9	---	---	**PT3b**	
		0	4.76%	0	2	---	---	**PT3c**	
		100%	100%	59	42	---	---	**Total**	
**0.926**		5.08%	4.76%	3	2	---	---	**Lymph node involvement**	
**0.944**		6.77%	7.14%	4	3	---	---	**Distant Metastasis**	
**0.066**		32.2%	14.28%	19	6	---	---	**1**	**Tumor Grade Group**
		30.5%	52.38%	18	22	---	---	**2**	
		16.94%	14.28%	10	6	---	---	**3**	
		1.69%	7.14%	1	3	---	---	**4**	
		18.64%	11.9%	11	5	---	---	**5**	
		100%	100%	59	42	---	---	**Total**	

*T-test and Chi-square test were performed on quantitative and qualitative data, respectively. Mortality was evaluated by Cox proportional Hazard test.

**Table 3:** ERG expression intensity depending on primary characteristics

P-value	Relative Frequency			Frequency			Mean ± SD				
	Strong expression	Moderate expression	Weak expression	Strong expression	Moderate expression	Weak expression	Strong expression	Moderate expression	Weak expression		
**0.696**	---	---	---	---	---	---	63.64±7.82	65.77±8.25	63.29±6.96	**Age**	
**0.493**	---	---	---	---	---	---	7.55±1.68	8.23±1.88	8.29±2.56	**Gleason score**	
**0.329**	---	---	---	---	---	---	19.35±18.09	13.41±6.83	11.61±6.45	**Primary PSA**	
**0.402**	0	0	0	0	0	0	---	---	---	**PT1a**	**Tumor Stage**
	0	0	0	0	0	0	---	---	---	**PT1b**	
	13.63%	7.69%	14.28%	3	1	1	---	---	---	**PT2a**	
	18.18%	0	0	4	0	0	---	---	---	**PT2b**	
	31.81%	69.23%	57.14%	7	9	4	---	---	---	**PT2c**	
	9.09%	0	0	2	0	0	---	---	---	**PT3a**	
	22.72%	23.07%	14.28%	5	3	1	---	---	---	**PT3b**	
	4.54%	0	14.28%	1	0	1	---	---	---	**PT3c**	
	100%	100%	100%	22	13	7	---	---	---	**Total**	
**0.385**	9.09%	0	0	2	0	0	---	---	---	**Lymph node involvement**	
**0.168**	0	15.38%	14.28%	0	2	1	---	---	---	**Distant Metastasis**	
**0.547**	66.66%	16.66%	16.66%	4	1	1	---	---	---	**1**	**Tumor Grade Group**
	68.18%	13.63%	18.18%	15	3	4	---	---	---	**2**	
	0	66.66%	33.33%	0	4	2	---	---	---	**3**	
	0	100%	0	0	3	0	---	---	---	**4**	
	60%	40%	0	3	2	0	---	---	---	**5**	

*T-test and chi-square test were performed on quantitative and qualitative data, respectively. Mortality was evaluated by Cox proportional Hazard test.

## Discussion

What we investigated in the present study was the association of ERG expression and its intensity with PC. In this study the specificities known as outcomes involve mortality, lymph node invasion, tumor's grade, and metastasis; however, we couldn't find a significant correlation between ERG expression and any of the elements mentioned above, which has been consistent with some of the studies, although there were conflicting results, too. 

In Yaskiv* et al.* study, high grade prostate neoplasia correlated with positive ERG expression and also all the samples negative for PC, were negative for ERG expression too (22). Verdu* et al.*, similar to our study, demonstrated no significant correlation between ERG expression and Gleason score, tumor invasion and lymph node involvement (21). Although Qi* et al.* used the same methods and couldn't find any correlation of age, disease stage, Gleason score or Ki67 index with ERG expression, but a remarkable association was obtained between ERG expression and preoperative PSA and mortality rate (23). In comparison to our study, Xu* et al.* found a significant link between ERG expression and lower age; though, in accordance with our study, Gleason score and disease stage manifested no affinity to ERG expression (24). In Bokhorst* et al.* survey, ERG expression level was incapable of predicting Gleason score ≥7 as a post-prostatectomy prognostic factor (25). Similar to our results, Pan* et al.* declared no relevance between ERG expression and age, Gleason score and even PSA; nevertheless, ERG expression showed a decreased level in the metastatic cases (26). In the study of Taris* et al.* in 2014, on cocaine race, ERG expression was associated with higher grades of PC as well as its metastatic form; albeit; this issue was not valid in the African race (11.5% vs 33%) (27). In the latest study of Koide* et al.* in 2019 regarding metastatic prostate cancer, no remarkable correlation was detected between ERG expression and two groups of incidental and metastatic patients (28). 

Summary of the alluded investigations suggests diverse and paradoxical results regarding the link between ERG expression and either mortality or PC extension in different societies. In fact, this relation vigorously is under the influence of demographic features, ethnic and genetic factors; therefore, employ-ment of ERG expression in PC outcomes prediction is favorable in some communities and on the other hand, inefficient in others.

In the present study, frequency of ERG expression in PC patients was 41.6% which has been so close to previous studies concerning this theme. Yaskiv* et al.* along with Verdu* et al.* reported the ERG expression rate in their studies as 42% and 49%, respectively; as it can be noted, the numbers were close to our result (21, 22). In contrast, He* et al.*, Qi* et al.*, Pan* et al.* and Bismar* et al.* attained much fewer percentages, like 15.5%, 23.2%, 15.4% and 28.2%, respectively (16, 23, 26, 29). Thus, the preceding studies, in general, displayed ERG expression involves a broad spectrum ranging from 15.4% to 49% in different societies, influenced by geographical and ethnic traits. 

Our study had some limitations. The study population was small and only included patients from Sina hospital, one of the main hospitals in Iran and other factors such as patient characteristics or antibody specifications interfered. Further studies including more cases and ancillary tests are necessary for introducing a prognostic factor. 

## Conclusion

In a nutshell, in our belief, evaluation of ERG expression and its intensity may have no essential role as an acceptable prognostic factor for anticipating whether prostate cancer itself or the outcomes accompanied in Iranian population. 

## Author Contributions

HMT and SMK were the principal investigator, project coordinator, and supervisor, LAS was the pathologist who checked the ERG expression professor who the surgery was under his supervision, AN wrote the manuscript. 
